# Phylogenetic analysis of TET2 gene variants in Pakistani acute myeloid Leukemia patients

**DOI:** 10.1093/bib/bbag058

**Published:** 2026-03-10

**Authors:** Mawada Elmagboul Abdalla Abakar, Mehad Almagboul Abdalla Abaker, Marco Antoniotti, Alex Graudenzi

**Affiliations:** Department of Informatics, Systems and Communication, University of Milan-Bicocca, Milan, 20126, Italy; Department of Molecular Medicine and Medical Biotechnology, University of Naples Federico II, Naples, 80131, Italy; Department of Informatics, Systems and Communication, University of Milan-Bicocca, Milan, 20126, Italy; Department of Informatics, Systems and Communication, University of Milan-Bicocca, Milan, 20126, Italy

## Abstract

**Aim:**

Acute myeloid leukemia (AML) is a heterogeneous malignancy caused by the proliferation of neoplastic myeloid progenitor cells. With mutations in TET2 playing a critical role in leukemogenesis and therapeutic response. Population-specific mutational patterns remain largely unexplored. Multiple sequence alignment and phylogenetic analysis was used to elucidate the evolutionary conservation and divergence of TET2 sequences in Pakistani AML patients providing insights into mutational hotspots and population-specific patterns.

**Methods:**

Twenty-five TET2 gene sequences (12 AML and 13 controls) from the Pakistani population were analyzed using multiple sequence alignment, distance matrix analysis, and phylogenetic tree construction using Geneious software (https://www.geneious.com/).

**Results:**

AML samples showed significant deletions in the catalytic domain (positions 693–876), essential for enzymatic function, and showed reduced sequence conservation than controls. Phylogenetic analysis revealed a distinct separation between AML and control clusters with high bootstrap values, indicating clear evolutionary divergence.

**Conclusions:**

Our findings revealed distinct TET2 mutational hotspots and evolutionary patterns that may inform biomarker development and targeted therapy strategies. These results motivate large-scale, diverse-population research to unravel the global effects of TET2 variations, and support the use of phylogenetics analysis in precision oncology.

**References:**

1. Q. Gao, K. Shen, and M. Xiao, ‘TET2 mutation in acute myeloid leukemia: biology, clinical significance, and therapeutic insights,’ Dec. 01, 2024. doi: 10.1186/s13148-024-01771-2.

2. Han, J. A., An, J., & Ko, M. (2015). Functions of TET proteins in hematopoietic transformation. *Molecules and cells*, 38(11), 925–935.

3. Schwartz, R., & Schäffer, A. A. (2017). The evolution of tumour phylogenetics: principles and practice. *Nature Reviews Genetics*, 18(4), 213–229.

